# Supplementary figures and images for: RNA-seq reveals Nup62 as a potential regulator for cell division after traumatic brain injury in mice hippocampus
Source: PeerJ. 2023 Mar 7;11:e14913. doi: 10.7717/peerj.14913 (PMC10000302; doi:10.7717/peerj.14913)

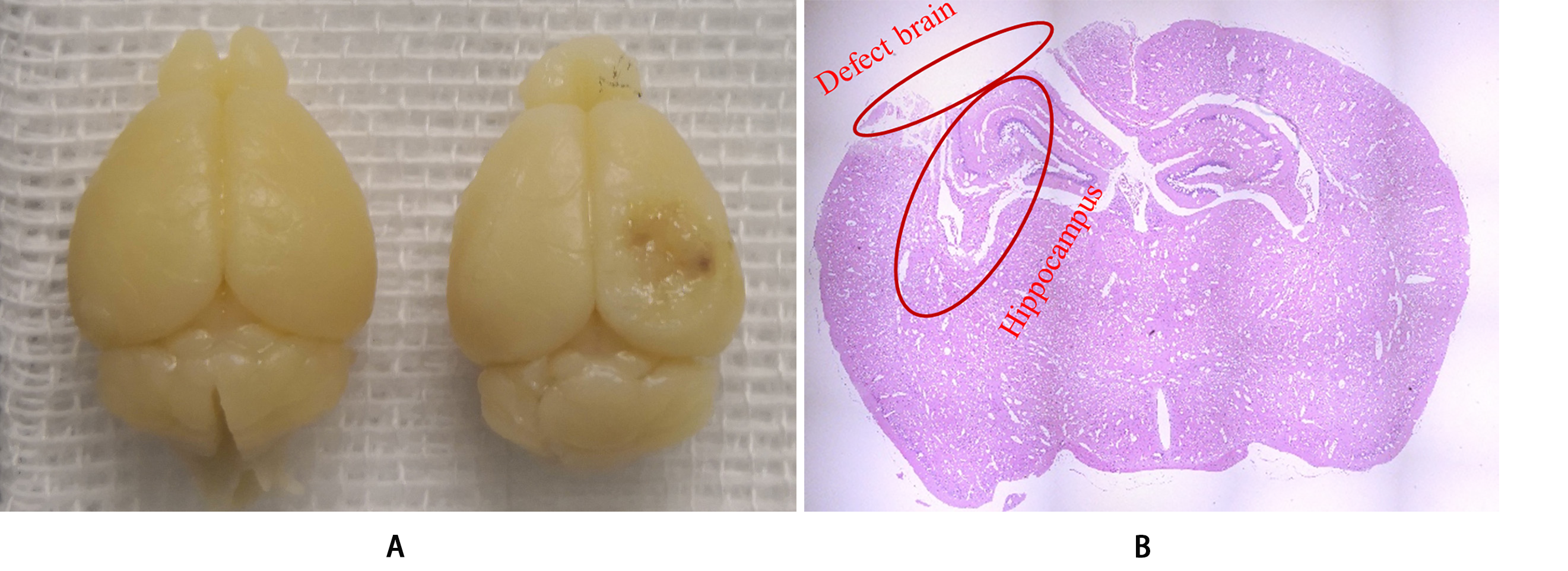

Supplement: Supplemental Information 6 — (A) whole brains in Sham and TBI. (B) HE stained section of the TBI hippocampus. [file peerj-11-14913-s006.png]

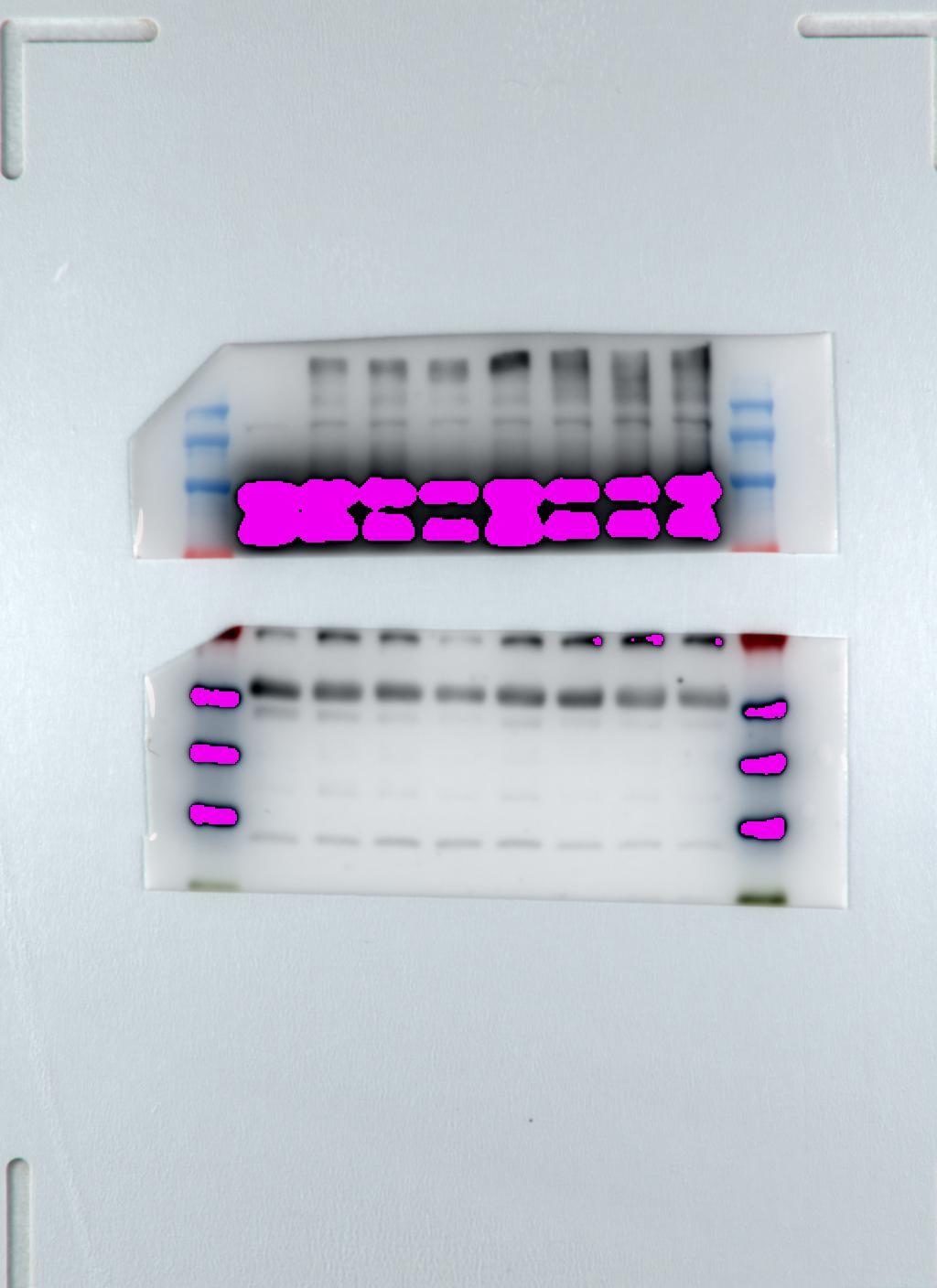

Supplement: Supplemental Information 7 [file peerj-11-14913-s007.jpg]
